# An Ultrasound-Guided Regional Anesthesia Elective for Emergency Medicine Residents

**DOI:** 10.21980/J8TP9B

**Published:** 2021-01-15

**Authors:** Ryan V Tucker, Robert Huang, William J Peterson, Brendan W Munzer, Molly Thiessen

**Affiliations:** * Michigan Medicine, Department of Emergency Medicine, Ann Arbor, MI; ^ Denver Health, Department of Emergency Medicine, Denver, CO

## Abstract

**Audience:**

This ultrasound-guided regional anesthesia elective is designed for emergency medicine residents.

**Length of Curriculum:**

The proposed length of this curriculum is over one week.

**Introduction:**

Ultrasound-guided regional anesthesia (UGRA) is a useful tool in the emergency department (ED) for managing painful conditions, and many programs have identified that these are useful skills for emergency providers; however, only about 53% of programs report teaching UGRA as part of their core curriculum, and there currently are no widely available or peer reviewed nerve block curricula designed for emergency medicine residents.

**Educational Goals:**

To deliver an immersive 1-week elective to provide residents a strong foundation in principles of UGRA and an introduction to 14 nerve block procedures applicable to care provided in the ED.

**Educational Methods:**

The educational strategies used in this curriculum include: instructional videos, written and online independent learning materials, one-on-one teaching at the bedside with an emergency ultrasound fellow, simulation of nerve block techniques using a femoral nerve block task trainer, and performance of supervised nerve block procedures on patients in the ED.

**Research Methods:**

All residents provided feedback through an online survey after completing the elective.

**Results:**

Eight residents completed the elective in the first year of implementation. Following completion of the UGRA curriculum, 8/8 (100%) of residents reported increased level of confidence in performing UGRA. In addition, 8/8 (100%) of residents reported they were “likely” or “very likely” to incorporate UGRA into their future EM practice. All 8 (100%) residents responded they were “very likely” to recommend the elective to other trainees. The elective received high ratings for overall quality with an average rating of 9.4 out of 10 (±0.7).

**Discussion:**

An elective in ultrasound-guided regional anesthesia can be successfully incorporated into an emergency medicine training program. The curriculum was successful in providing focused training in UGRA and resulted in increased resident confidence in performing nerve block procedures.

**Topics:**

Ultrasound-guided regional anesthesia, nerve block, resident, elective, pain.

## USER GUIDE

**Table:** 

List of Resources:	
Abstract	1
User Guide	3
Curriculum Chart	6
Appendix A: Ultrasound Guided Regional Anesthesia – Elective Syllabus	9
Appendix B: Nerve Block Matrix	12
Appendix C: Nerve Block Learning Resources	15
Appendix D: Elective Assessments Survey	20
Appendix E: Introduction to Ultrasound Guided Nerve Blocks	23
Appendix F: Femoral and Facia Iliaca Blocks	24
Appendix G: Forearm Nerve Blocks	25
Appendix H: Brachial Plexus Nerve Blocks	26
Appendix I: RAPTIR Block	27
Appendix J: Superficial Cervical Plexus Block	28
Appendix K: Axillary Deltoid Nerve Block	29
Appendix L: Serratus Anterior Plane Block	30
Appendix M: Transverse Abdominis Plane Block	31
Appendix N: Saphenous Nerve Block	32
Appendix O: Popliteal Sciatic Nerve Block	33
Appendix P: Posterior Tibial Nerve Block	34

**Learner Audience:**

Junior Residents, Senior Residents

**Length of Curriculum:**

1 week

**Topics:**

Ultrasound-guided regional anesthesia, nerve block, resident, elective, pain.

**Objectives:**

By the end of this elective learners will be able to:

1. Know the uses and indications for the 14 UGRA techniques described in this elective.
2. Describe the contraindications to performing nerve block procedures.
3. Identify the key anatomic landmarks for each nerve block procedure using ultrasound on models or patients in the ED.
4. Learn maximum allowable doses, duration of action, and uses for commonly used local anesthetic medications.
5. Recognize the signs and symptoms of local anesthetic systemic toxicity (LAST) as well as appropriate management of this condition and indications for Intralipid.

### Brief introduction

Effective pain management is a cornerstone of emergency medicine (EM) practice.^1^ EM physicians must utilize a variety of pain management techniques, including ultrasound-guided regional anesthesia (UGRA). Ultrasound-guided regional anesthesia utilizes real-time ultrasound guidance for needle placement near a peripheral nerve or in a fascial plane such that injection of anesthetic results in sensory blockade of a particular anatomic area. There are many potential advantages to using UGRA over conventional pain management techniques, including avoiding common and potentially dangerous side effects of opioid-based systemic analgesia and risks of procedural sedation.^2^ Ultrasound-guided regional anesthesia can provide effective short-term pain control to facilitate procedures such as reduction and splinting of fractures.^3^ The most well studied applications of UGRA in the Emergency Department (ED) are the femoral nerve and fascia iliaca blocks for acute hip fractures. In addition to effective pain control the femoral nerve block has been shown to reduce complications, such as pneumonia in elderly patients, and improve long-term functional outcomes.^4^

### Problem identification, general and targeted needs assessment

Despite the benefits of UGRA, many EM residents do not receive focused education in performing ultrasound-guided nerve blocks. In a study of residency programs in the United States, nearly all respondents believed that UGRA was a necessary skill for all EM physicians to learn;^5^ however, only 53% of residency training programs reported teaching UGRA as part of their core ultrasound curriculum. While a number of textbook and online resources exist, including a web-based tutorial for anesthesia residents^6^ and a recent review,^3^ there is no UGRA curriculum available to EM residents. Nearly all EM trainees in the United States complete a rotation dedicated to instruction in bedside ultrasound and attain basic ultrasound skills.^7^ Studies have shown that EM residents, after acquiring basic ultrasound skills, can perform individual nerve blocks effectively after minimal instruction.^8^ An elective specifically designed for EM trainees addresses this gap in training and ensures that future EM physicians are proficient in these important techniques.

The curriculum was designed as a 1-week elective rotation during the PGY2 through PGY4 year, after residents had completed a one-week introductory ultrasound rotation during their PGY1 year. A literature search was performed, open access UGRA resources were reviewed, including the New York School of Regional Anesthesia (NYSORA) website^9^ as well as the Highland Emergency Medicine Residency Ultrasound website.^10^ We compiled an extensive list of nerve block techniques and selected those most relevant to EM practice. An expert panel of ultrasound fellowship trained EM physicians reviewed the list of techniques and additional techniques were solicited. The panel was comprised of ultrasound experts from multiple institutions across the United States who practiced in both academic and community settings. They agreed on a final list of 14 techniques (Table 1).

### Goals of the curriculum

Provide an immersive elective educational experience for EM residents. Residents will focus on the key principles of UGRA and gain experience in 14 nerve blocks commonly performed in the ED.

### Objectives of the curriculum

By the end of this elective learners will be able to:

1. Know the uses and indications for the 14 UGRA techniques described in this elective.
2. Describe the contraindications to performing nerve block procedures.
3. Identify the key anatomic landmarks for each nerve block procedure using ultrasound on models or patients in the ED.
4. Learn maximum allowable doses, duration of action, and uses for commonly used local anesthetic medications.
5. Recognize the signs and symptoms of local anesthetic systemic toxicity (LAST) as well as appropriate management of this condition and indications for Intralipid.

### Educational strategies

The curriculum chart below details our educational strategies, content, learning objectives, intended learners, requirements, and targeted milestones.

### Results and tips for successful implementation

Before the start of the elective, we provided residents with the elective syllabus, a detailed table of all nerve block techniques covered in the course, and a list of print and online resources organized by nerve block technique. The elective educational experience included one-on-one teaching at the bedside with a fellow, simulation of nerve block technique using a femoral nerve block task trainer, and performing fellow-supervised nerve block procedures on patients in the ED. Each resident met with a fellow in the ED for a minimum of three one-on-one sessions, each session scheduled for 2 hours.

Between December 2018 and June 2019, 8 total residents completed the elective. Three PGY2 residents, 2 PGY3 residents and 3 PGY4 residents completed the elective. All residents provided feedback through an online survey after completing the elective. Data collection was given exempt status by our Institutional Review Board. The residents had minimal previous experience with UGRA. 7/8 (87.5%) residents had performed or directly observed 5 or fewer nerve block procedures before the elective. Following completion of the UGRA elective, 8/8 (100%) residents reported increased level of confidence in performing UGRA. 8/8 (100%) residents also reported they were “likely” or “very likely” to incorporate UGRA into their future EM practice. All residents responded they were “very likely” to recommend the elective to other trainees. The elective received high ratings for overall quality with an average rating of 9.4 out of 10 (±0.7). In free text responses, all residents cited a strength of the elective was one-on-one experience performing UGRA with the fellow. Four residents responded that more opportunities to perform ultrasound-guided nerve blocks on patients would improve the elective.

### Associated content

There are recorded video lectures for each nerve block procedure covered in the elective.

### Evaluation and feedback

Following initial implementation of the curriculum, we received feedback that residents desired more hands-on practice in performing nerve block procedures. We addressed this need by incorporating a femoral nerve block task trainer^11^ to simulate a nerve block procedure from start to finish. We also moved one-on-one scanning sessions in the ED from the morning to the afternoon and early evening when more opportunities to perform nerve block procedures on patients were available.

We also received feedback that the provided list of online resources could be supplemented with additional content for each nerve block procedure. In response, we recorded an introductory lecture, 5 to 15 minutes in duration, for each procedure and provided these to residents before their scheduled rotation. Residents reported in their evaluation surveys that these were a useful addition to the curriculum.

## Figures and Tables

**Table 1:** Nerve Block Procedures

Superficial Cervical Plexus Plane Block	Anterior Scalene Brachial Plexus Block
Supraclavicular Brachial Plexus Block	Infraclavicular Brachial Plexus Block
Axillary (Deltoid) Nerve Block	Serratus Anterior Plane Block
Radial Nerve Block	Median Nerve Block
Ulnar Nerve Block	Popliteal Sciatic Nerve Block
Saphenous Nerve Block	Posterior Tibial Nerve Block
Transversus Abdominis Plane Block	
Retroclavicular Approach to the Infraclavicular Region (RAPTIR) Brachial Plexus Block	

**Figure:**
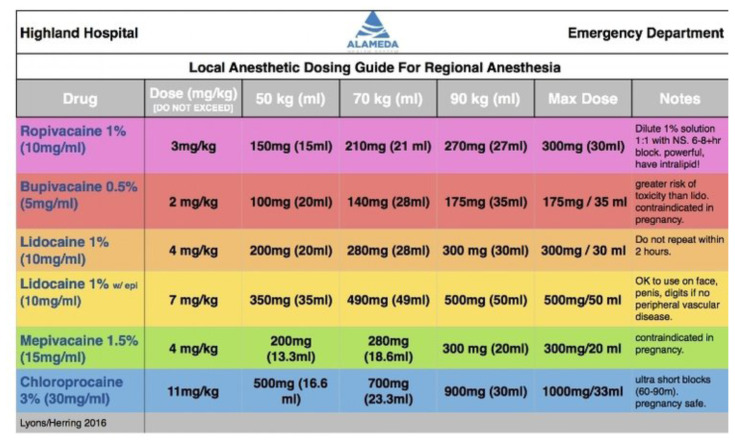
Highland EM Ultrasound Fueled Pain Management. http://highlandultrasound.com/med-guid

**Figure:**
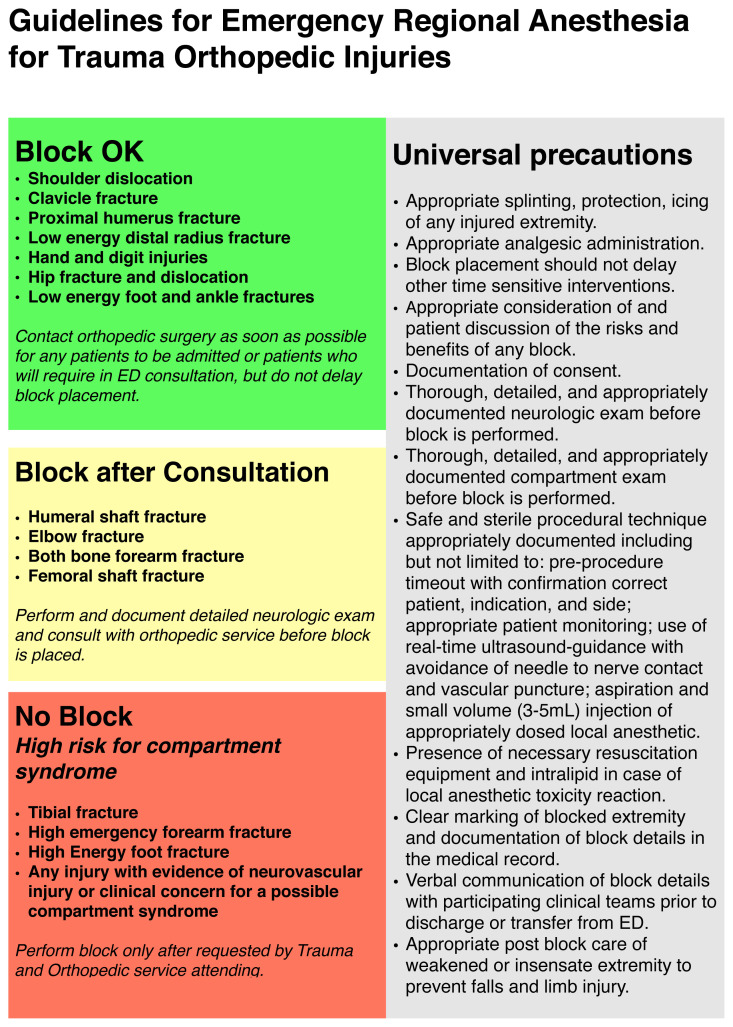
Guidelines for Emergency Regional Anesthesia for Trauma Orthopedic Injuries Highland EM Ultrasound Fueled Pain Management. http://highlandultrasound.com/med-guid

## DIDACTICS AND HANDS-ON CURRICULUM

**Table:** Curriculum Chart

Topic	Recommended Educational Strategy	Educational Content	Objectives	Learners	Timing, Resources Needed	Recommended Assessment, Milestones Addressed
Principles of Ultrasound-Guided Regional Anesthesia (UGRA)	Brief introductory lecture Elective Overview and Question and Answer session following lecture	-Performing UGRA safely -Potential benefits of nerve blocks to patients and providers -Indications and contraindicatio ns to nerve block procedures -Correct anesthetic dosing and calculation of maximum allowable dose -Local anesthetic systemic toxicity -Intralipid use and dosing -Sterile technique - Elective educational resources	The learner will receive an introduction to the elective curriculum structure, elective resources, and the key principles of performing UGRA	PGY2-PGY4	15 minutes (lecture) Instructors: 1 Equipment: PowerPoint and laptop/ screen 15 minutes (Overview and Q+A) Instructors:1 Equipment: Elective syllabus, Block Matrix, and Resources documents	Assessment: Learner demonstrates mastery of principles through direct observation of nerve block procedures on task trainer and patients
Femoral Nerve Block Simulation Session	Directly observed procedure simulation	- Necessary materials to perform nerve block procedure and where to obtain - Sterile technique - Needle tracking skills with ultrasound using long-axis technique	Learner will show proper technique in performing a nerve block procedure on a task trainer meant to simulate a femoral nerve block using sterile technique	PGY2-PGY4	45 minutes Instructors: 1 Equipment: femoral nerve block task trainer^11^, ultrasound machine, block needle/spin-al needle, syringes, sterile tubing, saline, sterile gloves, Chloraprepand sterile ultrasound probe cover	Assessment: Direct observation of procedure technique
One-on-One Scanning Sessions in the ED	Hands-on teaching at the bedside	- Identification of the key anatomic landmarks for each nerve block procedure - Optimal patient positioning for each procedure - Needle trajectory for each procedure	Learner will describe how to obtain ultrasound images for each nerve block procedure, how to position the patient for procedure success, and the optimal needle approach	PGY2-PGY4	2-hour sessions Instructors: 1 per 2 residents maximum Equipment: ultrasound machine, patients in the ED willing to consent to an educational ultrasound exam	Assessment: Direct observation of ultrasound images obtained with real-time feedback
Performance of Nerve Block Procedures in the ED	Hands-on ultrasound teaching at the bedside	- Selection of patients with indications for nerve blocks and no contra-indications - Proper informed consent process for nerve block procedures - Equipment selection, anesthetic volume and dose - Completing the nerve block procedure including troubleshooting methods - Discussion with consultants or admitting teams regarding risks and benefits of nerve block	Learner will demonstrate performance of an entire nerve block procedure on an ED patient, from patient selection to documentation of the procedure	PGY2-PGY4	2-hour sessions Instructors: 1 per 2 residents maximum Equipment: ultrasound machine, materials for nerve block procedure, ED patients with conditions with indications for nerve block procedure who are able to consent	Assessment: Direct observation of procedure performance with real-time feedback
Nerve Block Procedure Recorded Video Lectures	Introductory lectures covering each nerve block procedure	- Skin, muscular, and bony territory anesthetized by each nerve block procedure - Examples of common conditions for which each nerve block procedure would be useful - Ultrasound images and clips with important anatomy highlighted for identification - Photographs showing patient positioning, positioning of ultrasound probe, and needle approach - Animations showing ideal trajectory of needle to target - Description of the required anesthetic dose and volume needle to achieve adequate block - List of references for further reading	The learner will describe the anatomical area anesthetized, clinical uses, key anatomy on ultrasound images, optimal patient positioning and needle trajectory, and anesthetic dose/volume for each nerve block procedure	PGY2-PGY4	5 to 15 minutes for each lecture, 12 total lectures Equipment: computer with internet access	Assessment: Knowledge is tested during one-on-one scanning sessions by the instructor

### Appendix A: Ultrasound Guided Regional Anesthesia Elective

#### Description

This is a one-week elective providing instruction on ultrasound-guided nerve blocks useful to the emergency physician. Residents will complete a curriculum that includes independent study and hands-on learning of nerve block techniques. Ultrasound fellows and faculty will teach the elective.

#### Objectives

1. Learn the uses and indications for common ED blocks as well as the contraindications to performing these procedures.
2. Identify relevant anatomy using ultrasound for common ED nerve blocks.
3. Develop and improve skill/technique for ultrasound guided needle placement.
4. Learn maximum doses, duration of action, indications, and contraindications for commonly used local anesthetics, signs/symptoms of local anesthetic systemic toxicity (LAST), and appropriate treatment and indications for Intralipid.

#### List of selected nerve blocks to learn during elective

1. Femoral Nerve/Fascia Iliaca Block
2. Forearm Nerve Blocks (Median, Radial, Ulnar)
3. Brachial Plexus Nerve Blocks (Interscalene, Supraclavicular, Infraclavicular)
4. RAPTIR Nerve Block (Retroclavicular approach for infraclavicular brachial plexus)
5. Superficial Cervical Plexus Block
6. Axillary Nerve Block
7. Serratus Plane/Pectoralis Nerve Block
8. Transverse Abdominis Plane (TAP) Block
9. Saphenous Nerve Block
10. Popliteal Sciatic Nerve Block
11. Posterior Tibial Nerve Block

#### Activities to complete

- Independent study of nerve block procedures using below resources
- Identify anatomy using US in ED and review images with US fellow/faculty
- Rehearse nerve block procedure using models
- Perform nerve blocks if available in the ED

#### Resources

1. Website guide to quickly review a block procedure.
  - Highland Ultrasound: http://highlandultrasound.com/
2. In-depth Website guide to block procedures.
  - NYSORA – New York School of Regional Anesthesia: www.nysora.com
3. iBook Textbook Chapters: *Introduction to Bedside Ultrasound*
  - Volume 1 (Brachial Plexus Blocks – Interscalene, Supraclavicular, Infraclavicular, Axillary): https://itunes.apple.com/us/book/introduction-to-bedside-ultrasound/id554196012?mt=11
  - Volume 2 (Femoral, Forearm Blocks): https://itunes.apple.com/us/book/introduction-to-bedside-ultrasound/id647356692?mt=11
4. Short Instructional Videos – TAP, Superficial cervical plexus, Ulnar nerve, Popliteal sciatic nerve, Median nerve, Radial nerve, Supraclavicular brachial plexus, interscalene brachial plexus, posterior tibial nerve, Fascia Iliaca compartment block.
  - 5 Minute Sono: http://5minsono.com/vids/
5. Podcasts for specific blocks
  - Ultrasound Podcast - has a post on many of the above blocks: http://www.ultrasoundpodcast.com/?s=block
6. Block GuRu Lite – iPhone app reference, costs $6.99.

### Appendix B: Nerve Block Matrix – Ultrasound Guided Regional Anesthesia Elective

**Table:**

Block	Indications	Distribution	Probe Position	Volume
Interscalene Brachial Plexus	Shoulder dislocation, deltoid laceration (lac)/abscess, proximal/mid humerus fx	Shoulder, lateral arm, lateral forearm/hand	Transverse on neck, 3–4cm superior to clavicle, post. to internal jugular	7–15 mL
Supraclavicular Brachial Plexus	Distal humerus fx, elbow dislocation, lac/burn/abscess distal arm/forearm	Lateral arm, entire arm and hand distal to elbow	Transverse just superior to mid-clavicle	20–25 mL
Infraclavicular Brachial Plexus	Distal humerus fx, elbow dislocation, lac/burn/abscess distal arm/forearm	Lateral arm, entire arm and hand distal to elbow	Inferior to clavicle, sagittal, just medial to coracoid process	25–35 mL
Retroclavicular approach for Infraclavicular Brachial Plexus (RAPTIR)	Distal humerus fx, elbow dislocation, lac/burn/abscess distal arm/forearm	Lateral arm, entire arm and hand distal to elbow	Inferior to clavicle, sagittal, just medial to coracoid process	25–35 mL
Deltoid	Abscess/lac over the deltoid	Lateral shoulder	Sagittal, posterior arm 4cm inferior to the acromion	5–10 mL
Median	Lac medial palm, fx 2^nd^ or 3^rd^ digits	Palmar hand, medial 1^st^ digit through lateral 4^th^ digit	Transverse, middle of forearm	2–5 mL
Ulnar	Lac medial hand, fx 4^th^ or 5^th^ digits	Medial hand	Transverse on medial forearm, identify ulnar artery and trace proximal	2–5 mL
Radial	Lac posterior hand, fx 1^st^ through 4^th^ digits	Volar hand, 1^st^ through medial 4^th^ digit	Transverse on lateral arm, identify artery and trace proximal/Transverse proximal to lateral epicondyle	2–5 mL
Femoral/Fascia Iliaca	Femur fx	Hip, medial thigh, medial leg, medial ankle/foot	Transverse in femoral crease	10–30 mL
Saphenous	Lac or abscess on medial leg or foot	Medial distal thigh through medial foot	Transverse on anteromedial thigh	5–10 mL
Popliteal Sciatic	Fx distal tib/fib, lac or abscess lower leg	Lower leg/ankle/foot, excluding medial leg/ankle	Transverse of popliteal fossa, trace proximally	15 – 20 mL
Posterior Tibial	Lac to sole of foot, FB in sole of foot	Majority of sole of the foot excluding extreme medial and lateral	Transverse, just proximal to medial malleolus, trace proximally	3 – 5 mL
Superficial cervical plexus (Plane Block)	Lac lower ear, central line placement, distal clavicle fx, lac/abscess anterolateral neck	Anterolateral neck, anteauricular and retroauricular areas, skin overlying clavicle	Transverse over midpoint of SCM (sternocleidomastoid) muscle (posterior border)	5 – 15 mL
Serratus Anterior (Plane Block)	Rib fx, chest tube placement	Anterolateral chest, T3–T9 dermatomes	Sagittal, between 4^th^ and 5^th^ ribs, anterior axillary line	20 – 30 mL
Transversus Abdominis (Plane Block)	Abdominal wall abscess, abdominal wall lac	Hemi-abdomen, T10–T12 dermatomes	Transverse, midaxillary line, proximal to iliac crest, lower abdomen	20 – 30 mL

### Appendix C: Ultrasound Guided Regional Anesthesia Elective Resource List

1. Interscalene Brachial Plexus
  - NYSORA - https://www.nysora.com/techniques/upper-extremity/intescalene/ultrasoundguided-interscalene-brachial-plexus-block/
  - Introduction to Bedside Ultrasound: Chapter 11
  - 5 Min Sono - http://5minsono.com/is/
2. Supraclavicular Brachial Plexus
  - NYSORA – https://www.nysora.com/regional-anesthesia-for-specific-surgical-procedures/upper-extremity-regional-anesthesia-for-specific-surgical-procedures/anesthesia-and-analgesia-for-elbow-and-forearm-procedures/ultrasound-guided-supraclavicular-brachial-plexus-block/
  - Introduction to Bedside Ultrasound: Chapter 11
  - 5 Min Sono - http://5minsono.com/supraclav/
3. Infraclavicular Brachial Plexus
  - NYSORA - https://www.nysora.com/techniques/upper-extremity/infraclavicular/
  - Introduction to Bedside Ultrasound: Chapter 11
4. Retroclavicular approach to Infraclavicular Brachial Plexus (RAPTIR)
  - Highland Ultrasound - http://highlandultrasound.com/raptir/
  - Review Paper - Luftig J, Mantuani D, Herring AA, Nagdev A. Ultrasound-guided retroclavicular approach infraclavicular brachial plexus block for upper extremity emergency procedures. AJEM. 2017; 35(5): 773–777.
  - ACEP Now - https://www.acepnow.com/article/how-to-effectively-block-an-acutely-fractured-distal-radius/
5. Axillary
  - Highland Ultrasound - http://highlandultrasound.com/axillary-nerve-delt/
6. Median
  - Highland Ultrasound - http://highlandultrasound.com/forearm-blocks/
  - Introduction to Bedside Ultrasound: Chapter 21, Section 3
  - 5 Min Sono - http://5minsono.com/mnb/
7. Ulnar
  - Highland Ultrasound - http://highlandultrasound.com/forearm-blocks/
  - Introduction to Bedside Ultrasound: Chapter 21, Section 3
  - 5 Min Sono - http://5minsono.com/unb/
8. Radial
  - Highland Ultrasound - http://highlandultrasound.com/forearm-blocks/
  - Introduction to Bedside Ultrasound: Chapter 21, Section 3
  - 5 Min Sono - http://5minsono.com/rnb/
9. Femoral/Fascia Iliaca
  - NYSORA - https://www.nysora.com/techniques/lower-extremity/ultrasound-guided-femoral-nerve-block/
  - Introduction to Bedside Ultrasound: Chapter 20
  - Highland Ultrasound - http://highlandultrasound.com/femoral-block/
10. Saphenous
  - Highland Ultrasound - http://highlandultrasound.com/saphenous-block/
  - NYSORA - https://www.nysora.com/regional-anesthesia-for-specific-surgical-procedures/lower-extremity-regional-anesthesia-for-specific-surgical-procedures/foot-and-anckle/ultrasound-guided-saphenous-subsartorius-adductor-canal-nerve-block/
11. Popliteal Sciatic
  - 5 Min Sono - http://5minsono.com/pop/
  - NYSORA - https://www.nysora.com/regional-anesthesia-for-specific-surgical-procedures/lower-extremity-regional-anesthesia-for-specific-surgical-procedures/foot-and-anckle/ultrasound-guided-popliteal-sciatic-block/
12. Posterior Tibial
  - 5 Min Sono - http://5minsono.com/ptnb/
  - Highland Ultrasound - http://highlandultrasound.com/posterior-tibial-block/
13. Superficial Cervical Plexus Plane
  - 5 Min Sono - http://5minsono.com/scp/
  - Highland Ultrasound - http://highlandultrasound.com/superficial-cervical-plexus-block/
14. Serratus Anterior Plane
  - NYSORA - https://www.nysora.com/regional-anesthesia-for-specific-surgical-procedures/thorax/pectoralis-serratus-plane-blocks/
  - Highland Ultrasound - http://highlandultrasound.com/rib-fractures/
15. Transversus Abdominis Plane (TAP)
  - 5 Min Sono - http://5minsono.com/tap/
  - NYSORA - https://www.nysora.com/regional-anesthesia-for-specific-surgical-procedures/abdomen/ultrasound-guided-transversus-abdominis-plane-quadratus-lumborum-blocks/

### Appendix D: Elective Assessment – Ultrasound Guided Regional Anesthesia Elective

#### Consent

By completing this assessment, you agree to be enrolled in a study to assess the effectiveness of the elective. You do not have to complete this assessment and have the right to refuse participation. Refusing to participate will in no way affect your standing in the residency. No personal information will be collected, and responses are anonymous. If you have questions, contact XXX, MD at XXX@XXX. All feedback on how to improve the elective experience is greatly appreciated.

I Agree
I Decline

1. What is your current level of training?
  EM1
  EM2
  EM3
  EM4
2. Before completing the elective, how many nerve block procedures had you performed or directly observed?
  0
  1–3
  3–5
  5 or more
3. Before completing the elective, what was your level of confidence in performing nerve block procedures?
  Extremely confident
  Very confident
  Somewhat confident
  Not so confident
  Not at all confident
4. After completing the elective, what is your level of confidence in performing nerve block procedures?
  Extremely confident
  Very confident
  Somewhat confident
  Not so confident
  Not at all confident
5. How likely are you to recommend this elective to other residents?
  Extremely likely
  Somewhat likely
  Neither likely nor unlikely
  Somewhat unlikely
  Extremely unlikely
6. Please rate the elective experience overall.
  1. 1 – Poor
  2. 2
  3. 3
  4. 4
  5. 5 – Good
  6. 6
  7. 7
  8. 8
  9. 9
  10. 10 – Excellent
7. What are the most positive aspects of the elective experience?
8. How can the elective experience be improved?
9. Please provide any additional feedback on the elective.

Thank you very much for completing this elective assessment.

### Appendix E: Introduction to Ultrasound Guided Nerve Blocks

Please see associated lecture Lecture Link: https://youtu.be/Bp9EwDXKK_A

### Appendix F: Femoral and Facia Iliaca Blocks

Please see associated lecture Lecture Link: https://youtu.be/9FnS0ZOvh6o

### Appendix G: Forearm Nerve Blocks

Please see associated lecture Lecture Link: https://youtu.be/4wTISU4156I

### Appendix H: Brachial Plexus Nerve Blocks

Please see associated lecture Lecture Link: https://youtu.be/TOrKoTcuZc8

### Appendix I: RAPTIR Block

Please see associated lecture Lecture Link: https://youtu.be/pZTIwxZ5E4E

### Appendix J: Superficial Cervical Plexus Block

Please see associated lecture Lecture Link: https://youtu.be/siGBeisWC1Q

### Appendix K: Axillary Deltoid Nerve Block

Please see associated lecture Lecture Link: https://youtu.be/_2TBXobcH1A

### Appendix L: Serratus Anterior Plane Block

Please see associated lecture Lecture Link: https://youtu.be/E_q1yVADE6Q

### Appendix M: Transverse Abdominis Plane Block

Please see associated lecture Lecture Link: https://youtu.be/JbY-7aT8m7I

### Appendix N: Saphenous Nerve Block

Please see associated lecture Lecture Link: https://youtu.be/7HwnQonE5ok

### Appendix O: Popliteal Sciatic Nerve Block

Please see associated lecture Lecture Link: https://youtu.be/lH1u6oPt4nY

### Appendix P: Posterior Tibial Nerve Block

Please see associated lecture Lecture Link: https://youtu.be/3mtXyF04BC4
